# Layered Composites for High Tan Delta Plateau over Wide Temperature Range

**DOI:** 10.3390/polym16243587

**Published:** 2024-12-22

**Authors:** Taoxi Wang, Hongmei Chen, Wei Jun Liang, Boon Siang Lucas Ng, Runzhi Lu, Ji Qi, Huaquan Wang, Junhua Zhang, Hui Xie, Rui Xiao, Wei Min Huang

**Affiliations:** 1 State Key Laboratory of Mechanics and Control for Aerospace Structures, Nanjing University of Aeronautics and Astronautics, 29 Yudao Street, Nanjing 210016, China; 2 College of Chemistry and Materials Science, Sichuan Normal University, Chengdu 610066, China; chenhongmei@sicnu.edu.cn; 3 School of Mechanical and Aerospace Engineering, Nanyang Technological University, 50 Nanyang Avenue, Jurong West 639798, Singapore; wliang006@e.ntu.edu.sg (W.J.L.); lng040@e.ntu.edu.sg (B.S.L.N.); n2409308j@e.ntu.edu.sg (R.L.); qiji0003@e.ntu.edu.sg (J.Q.); 4 China Resources Cement Technology Research and Development Co., Ltd., Guangzhou 510460, China; whq931@163.com; 5 State Key Laboratory of Polymer Materials Engineering, Polymer Research Institute of Sichuan University, Chengdu 610065, China; zhangjh@scu.edu.cn; 6 Key Laboratory of Advanced Technologies of Materials, Ministry of Education, School of Materials Science and Engineering, Southwest Jiaotong University, Chengdu 610031, China; huixie@swjtu.edu.cn; 7 Key Laboratory of Soft Machines and Smart Devices of Zhejiang Province, Department of Engineering Mechanics, Zhejiang University, Hangzhou 310027, China; rxiao@zju.edu.cn

**Keywords:** Tan Delta plateau, phase transition, elastic layer, storage modulus

## Abstract

Tan Delta reflects the viscoelastic behavior of materials, particularly polymers. In most cases, a high Tan Delta value is associated with transitions (such as glass transition or melting), enabling effective damping properties near these temperature ranges. However, achieving a high Tan Delta over a broad temperature range is challenging, particularly for engineering applications that involve significant temperature fluctuations. This paper presents a straightforward method using layered composites, where a polymer layer is sandwiched between two highly stretchable elastic fabrics, to achieve a wide Tan Delta plateau (TDP) across a broad temperature range. The three-layer configuration consists of a polymer core embedded between two elastic layers. All samples prepared with this architecture consistently exhibit the TDP. Further investigations examine the influence of factors such as the number of layers and the stretchability of the elastic fabrics. The results demonstrate that the TDP can be effectively tailored for engineering applications using this layered design.

## 1. Introduction

Tan Delta (tan δ) represents the ratio of a material’s viscous response to its elastic response in viscoelastic behavior, i.e.,

(1) $$\mathrm{Tan}\;\;\mathrm{Delta}=\frac{E^{\prime \prime }}{E^{\prime }}$$

where E″ and E′ represent the loss modulus and storage modulus of a material, respectively. E″ quantifies a material’s capacity to dissipate energy during deformation, reflecting its viscous or liquid-like behavior [1]. In contrast, E′ measures a material’s ability to store energy elastically under deformation, serving as a critical indicator of its solid-like behavior [2]. Tan Delta is usually measured by, for instance, a dynamic mechanical analysis (DMA) test [3]. A high Tan Delta plateau (TDP) over a wide temperature range normally indicates the outstanding damping property of a material even when the working environmental temperature fluctuates dramatically within a temperature range [4], which is highly in demand in many engineering applications [5,6,7,8].

**Figure 1:**
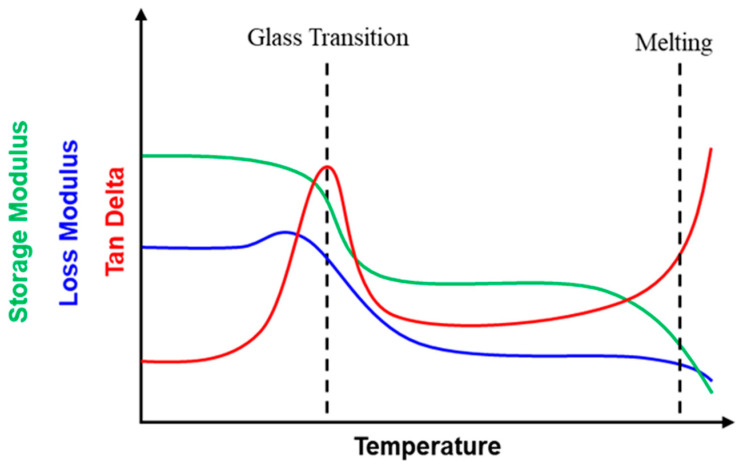
Illustration of a typical DMA thermogram of polymers.

For polymers, their viscoelasticity is mostly determined by the glass transition or melting/crystallization [9]. Consequently, as illustrated in Figure 1, the Tan Delta peak appears at around the glass transition temperature (T_g_) or the melting/crystallization temperature (T_m_/T_c_). A TDP was only reported in a couple of polymers, such as polypropylene and its composites, while the corresponding Tan Delta value in the TDP range is low (≈ 0.1 to 0.15) [10,11,12,13]. It is more common to introduce multiple materials corresponding to multiple Tan Delta peaks within the required temperature range to approximate a TDP. Since multiple materials are used, a high Tan Delta value within a TDP is practically not achievable.

Recently, a TDP (Tan Delta > 0.5) from 65 °C to 105 °C was found in a vitrimer-like polyurethane (PU) upon heating in [14,15]. Vitrimer is a newly coined term for polymers featuring reversible links. Consequently, vitrimers represent a novel polymer category situated between thermoplastics and thermosets. The underlying mechanism behind this interesting feature of the TDP in this vitrimer-like PU is explained as the result of the gradual cross-linking dissociation of its covalent adaptable network (CAN) [16]. Thus, while the material is able to largely maintain its shape before the CAN is completely removed [17], Tan Delta increases to a relatively higher value upon heating to its melting temperature and then remains until the dissociation process is complete [15]. Consequently, a TDP is virtually observed. As mentioned in [14], such a TDP phenomenon is only achievable in a vitrimer-like material with certain conditions. Furthermore, this kind of vitrimer-like material is very soft within the temperature range of the TDP, and therefore it cannot maintain its shape during thermal fluctuations. Thus, its real engineering application is limited.

Inspired by the working mechanism reported in [14], it is expect that the TDP phenomenon may be archivable in a composite of a highly elastic material and a highly viscous material, where the high viscosity may appear within a particular temperature range, for instance, above the crystallization temperature upon heating to fully melting. As noted in [14,15], the presence of a very weak network (characterized by a low E′) is a prerequisite for achieving a TDP, which restricts its applicability to certain specialized materials. Consequently, the use of a TDP in practical engineering scenarios remains highly constrained.

This paper introduces a novel structuring approach, referred to as layered composites, where a polymer layer is sandwiched between two pieces of highly stretchable soft fabric (low E′) in a three-layer configuration. This method enables the creation of a TDP across a broad range of materials. Such a straightforward structuring strategy holds promise for providing innovative solutions to various engineering challenges. Various polymer types characterized by a high E″ value, including vitrimer-like, thermoplastic, and shear-thickening polymers, are utilized for a systematic investigation. Subsequent parametric studies are undertaken with the objective of elucidating the fundamentals.

The outline of this paper is as follows. Section 2 is about the materials used and experiments carried out. The results and analysis are presented in Section 3. In Section 4, additional experiments are discussed to unveil the underlying mechanisms and compare performance across various configurations. Section 5 provides a summary of the main conclusions from this study, which are mainly experimental, along with an overview of the primary challenges anticipated in the future.

## 2. Materials and Methods

A vitrimer-like polyurethane (vitrimer, TPUA-123-H1111A from Taiwan PU Corporation (Taipei, Taiwan), which is very similar to another vitrimer-like polyurethane, TPU 262A [15], provided by the same company), a thermoplastic polyurethane (TPU, TPU 265A from Taiwan PU Corporation, Taiwan [18]), an ethylene-vinyl acetate (EVA, Cosmothene H2181 from Polyolefin Company (Singapore) Pte Ltd. [19]), a self-prepared D3O [20]-like shear-thickening material (STM), and Bostik 801103 Blu Tack adhesive (Blu Tack, available from https://www.amazon.sg/) were used.

A typical commercial-two directionally stretchable polyester fabric (spandex) (thickness: 0.5 mm) was used as the elastic layer. A typical commercial-one directionally stretchable polyester fabric (thickness: 0.7 mm) was also used for comparison.

**Figure 2:**
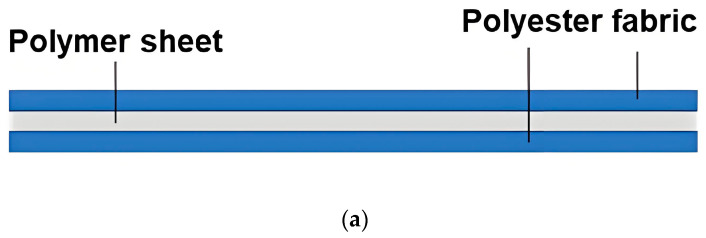

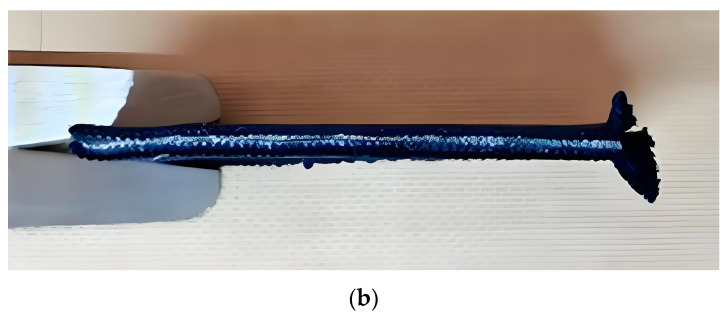
Illustration (**a**) and photo (**b**) of three-layered laminated sample.

In the process of fabricating layered samples, the polymer (vitrimer, TPU, EVA, STM, or Blu Tack) was firstly hot-compressed into a 1 mm-thick sheet at well above its melting point (if applicable), or compressed at room temperature (about 25 °C) into a 1 mm-thick sheet, if it is easy to deform at room temperature. Hot compression or room temperature compression was repeated one more time with two pieces of polyester fabrics on the top and bottom of the polymer sheet to create strong bonding between layers. After air-cooling to the room temperature, a rectangular sample of 3 cm × 12 cm was cut out (along the stretchable/non-stretchable direction) for a DMA test. Refer to Figure 2 for an illustration of the laminated architecture (Figure 2a) and the actual piece of a three-layered laminated sample (Figure 2b).

A DSC Q200 machine from TA Instruments (New Castle, DE, USA) with nitrogen gas for cooling was used to carry out the differential scanning calorimetry (DSC) test on small pieces cut from the as-received vitrimer, TPU, EVA, STM, and Blu Tack to reveal their corresponding transition temperatures. All DSC tests were conducted with a constant temperature ramp rate of 10 °C/min, including a three-minute holding period during each heating/cooling transition. Two consecutive cycles were performed to account for potential effects of prior thermal history on the results of the first cycle. The maximum and minimum temperatures during thermal cycling were adjusted based on the specific material being tested.

A Shimadzu AG-10kNXplus STD (Shimadzu, Kyoto, Japan) universal testing machine was used to stretch the polyester fabrics (sample size: 60 mm × 5 mm) to 20% strain at a strain rate of 10^−3^/s to obtain the stress-versus-strain relationships along two directions (stretchable/non-stretchable). A small preload of 0.008 MPa was applied. Herein, the stress and strain mean the engineering stress and engineering strain, respectively.

A Discovery DMA 850 machine also from TA Instruments (New Castle, DE, USA) with a heating/cooling speed control function was used in all the DMA tests in film tension mode. A constant frequency of 1 Hz was applied and the oscillation strain was 0.1%. Thermal cycling as detailed in Table 1 was carried out in each test. Note that the laminated STM sample was cooled to −10 °C instead of −30 °C in the last cycle. In addition to laminated samples, two pure polymer sheet (i.e., without elastic fabric) samples, namely pure vitrimer and pure EVA, were also tested in a similar way for comparison.

**Figure 3:**
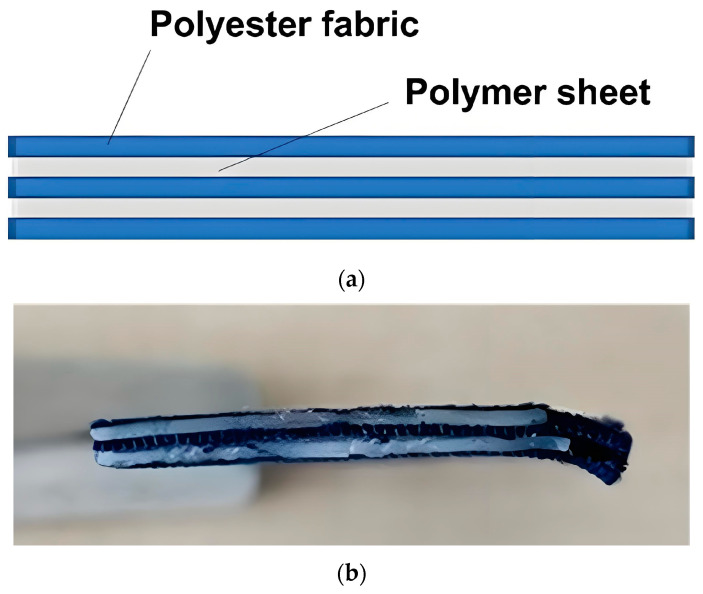
Illustration (**a**) and photo (**b**) of five-layered laminated vitrimer sample.

A five-layered laminated vitrimer sample was also prepared in a similar way. Refer to Figure 3a,b for its architecture (illustration) and a photo of the actual sample, respectively. The thickness of the polymer layer is the same as that of the three-layered samples.

It should be noted that these selected polymers bond very well with the elastic spandex after the manufacturing process. No delamination was observed in all DMA tests.

## 3. Results

### 3.1. DSC

**Figure 4:**
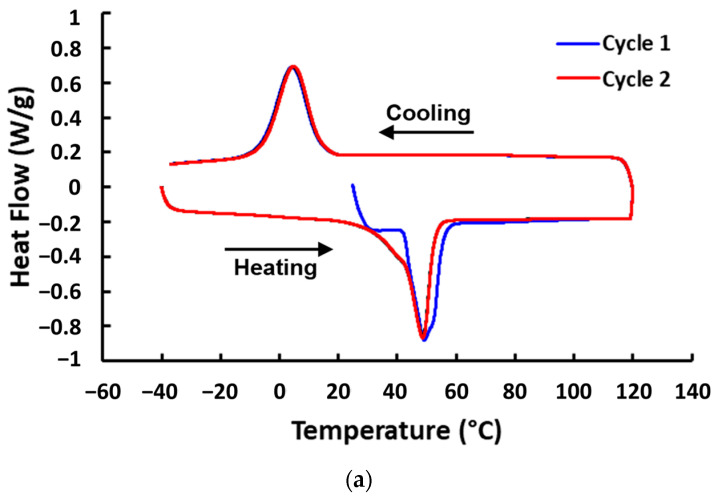

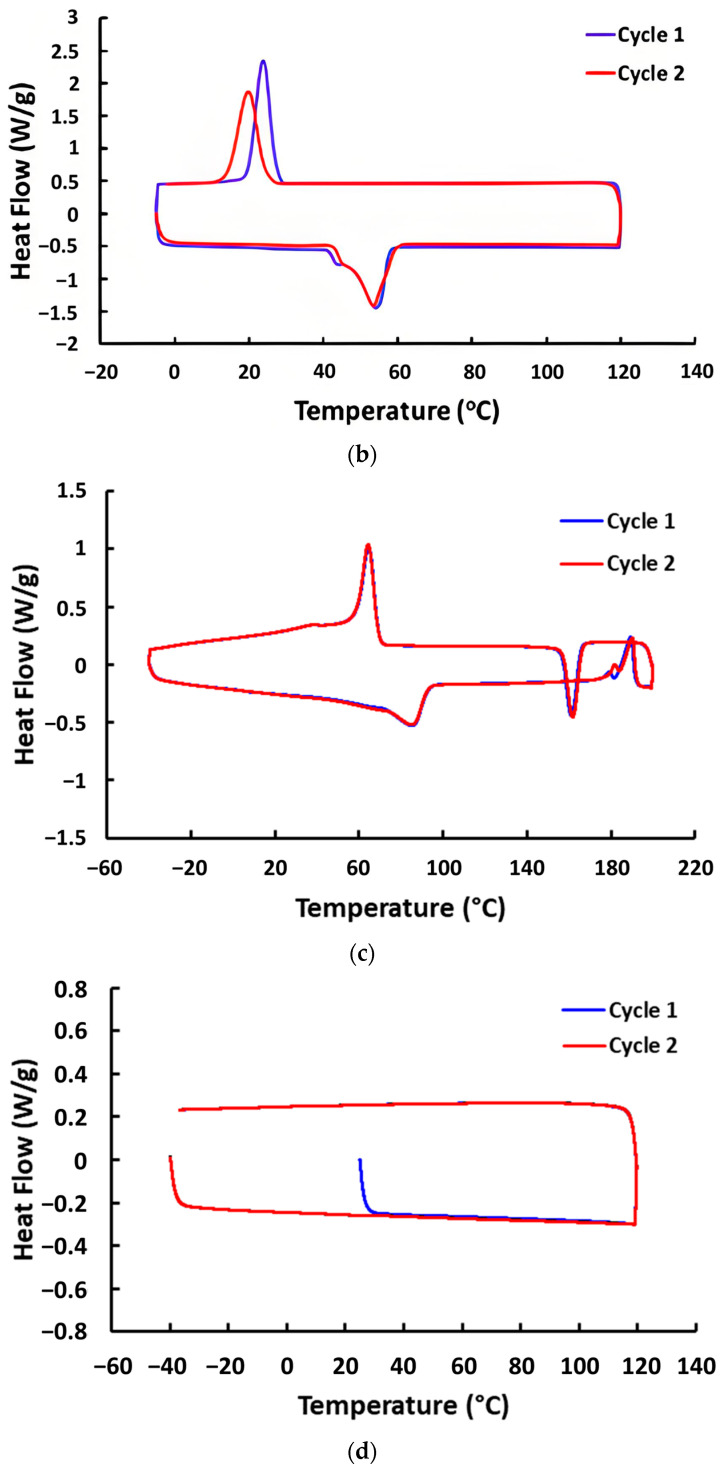

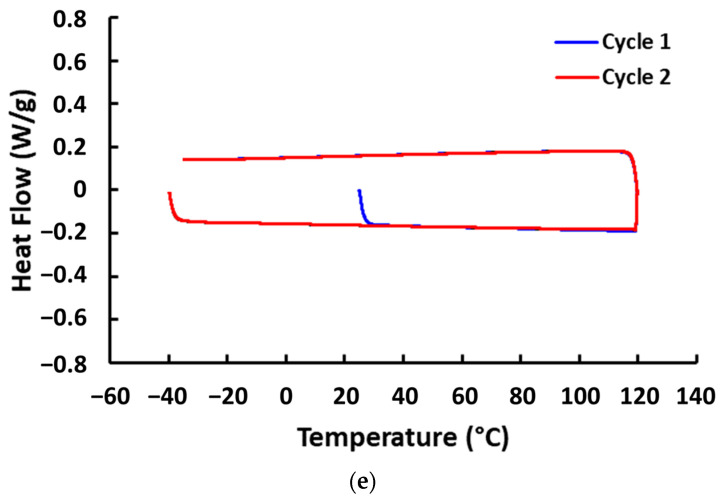
DSC results of (**a**) vitrimer, (**b**) TPU, (**c**) EVA, (**d**) STM, and (**e**) Blu Tack.

The DSC results of all tested polymeric materials are plotted in Figure 4. In Figure 4a–c, melting and crystallization upon heating and cooling, respectively, in vitrimer, TPU, and EVA, can be clearly observed. However, while TPU and EVA are thermoplastic, so that they are able to flow easily upon heating to above the melting temperature, this type of vitrimer does not flow at all upon heating to its melting temperature. Only at much higher temperatures, where the reversible cross-linking is fully removed, vitrimer becomes easy to flow like normal thermoplastics (refer to [15]). As for STM and Blu Tack (Figure 4d,e), no apparent transition can be observed based on the heat flow-versus-temperature curves.

### 3.2. Mechanical Tests

**Figure 5:**
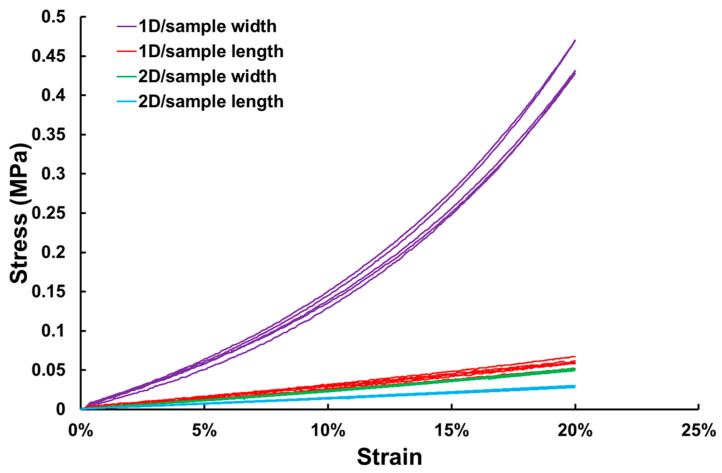
Typical engineering stress-versus-engineering strain relationships of two-directionally (2D) and one-directionally (1D) stretchable polyester fabrics upon uniaxial tension to 20% strain.

The stress-versus-strain relationships upon uniaxial tension to 20% strain along the stretchable/non-stretchable directions of the two-directionally and one-directionally stretchable polyester fabrics are plotted in Figure 5. Each type of test was repeated five times using different samples. Sample length and width directions correspond to the two stretchable directions or stretchable and non-stretchable directions, respectively, of the original fabric. As shown, the so-called non-stretchable direction is not exactly non-stretchable for the tested fabric, but the stress increases dramatically upon stretching when compared with that of the stretchable direction. As for the stress-versus-strain relationship along the stretchable direction, the increase in stress upon stretching is much more gradual, and there is not much difference in the stress-versus-strain relationship in both one-directionally and two-directionally stretchable polyester fabrics.

### 3.3. Tan Delta

**Figure 6:**
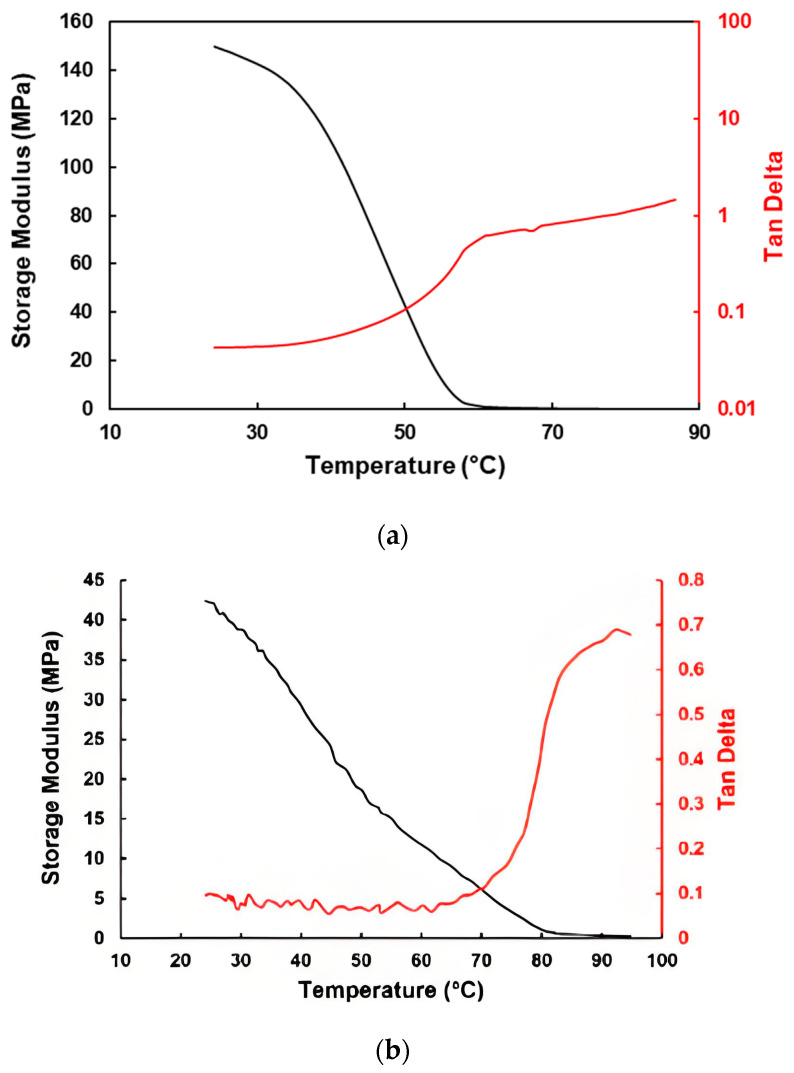
Storage modulus and Tan Delta of pure vitrimer (**a**) and EVA (**b**) upon heating.

In Figure 6, the storage modulus- and Tan Delta-versus-temperature relationships upon heating of pure vitrimer and pure EVA (a typical thermoplastic polymer) are presented to show the difference between the vitrimer and thermoplastic. Upon heating to the melting transition range, thermoplastic EVA becomes easy to flow, so that the corresponding Tan Delta increases continuously, while for the vitrimer, as the reversible cross-linking gradually disappears upon further heating, the corresponding Tan Delta increases slowly to virtually form a kind of plateau, until there is not any more reversible cross-linking and thus the vitrimer becomes easy to flow like normal thermoplastics [14].

**Figure 7:**
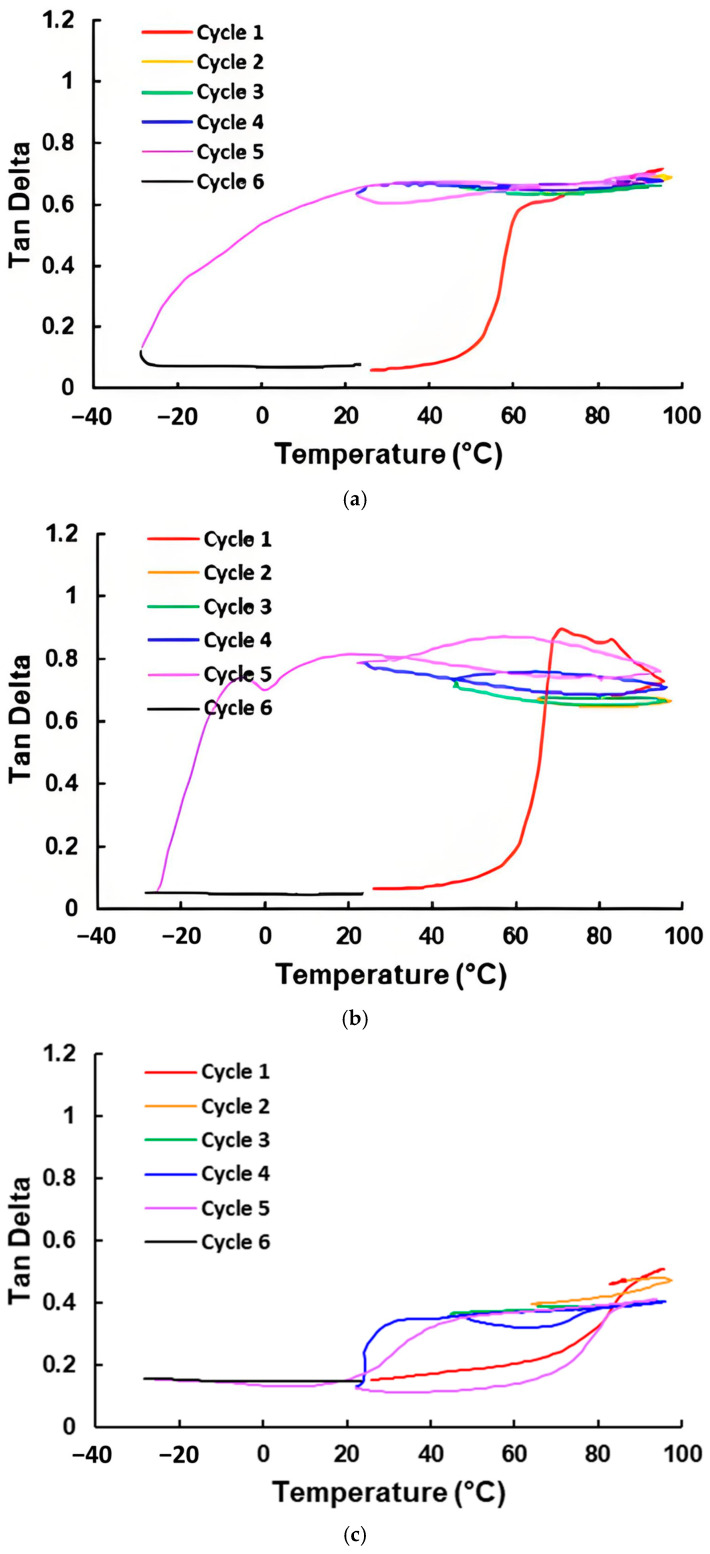

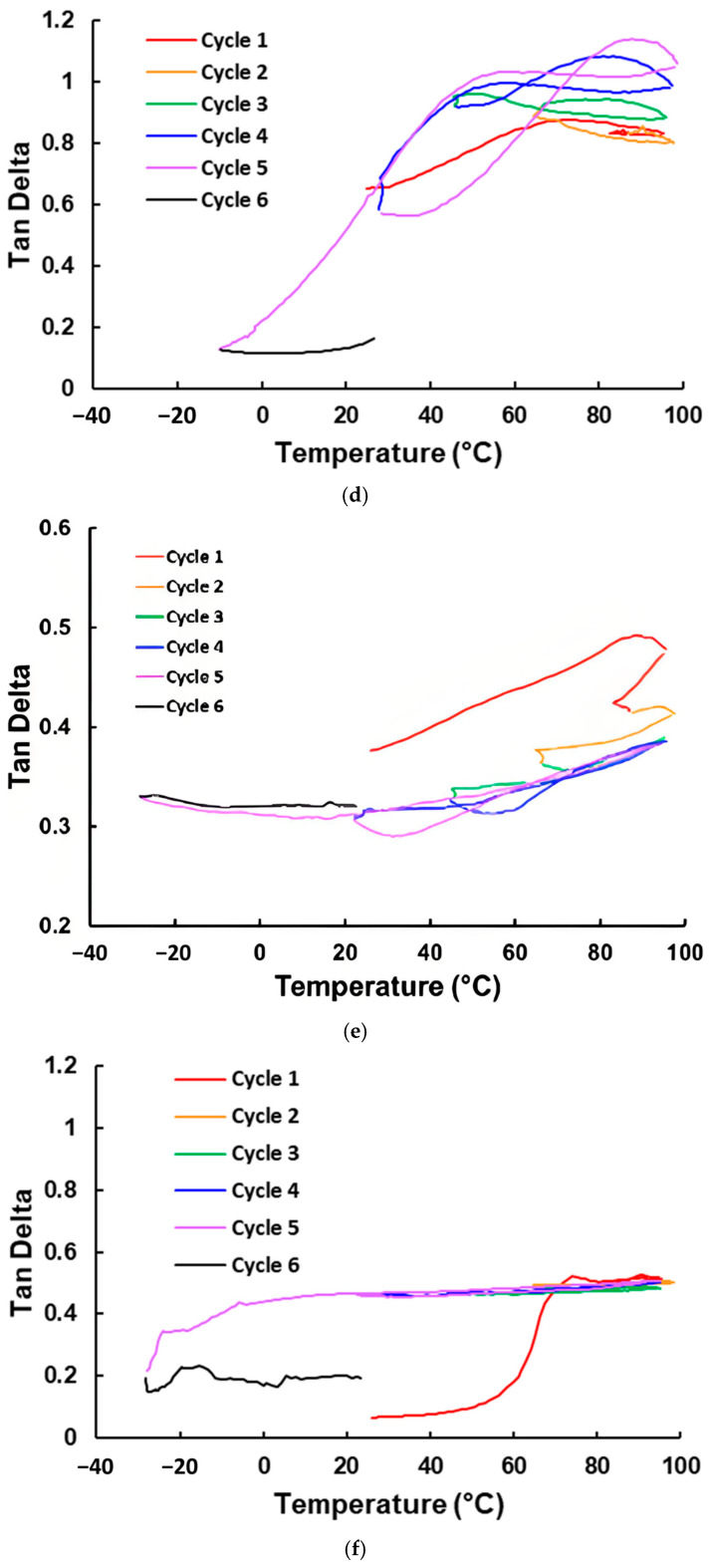

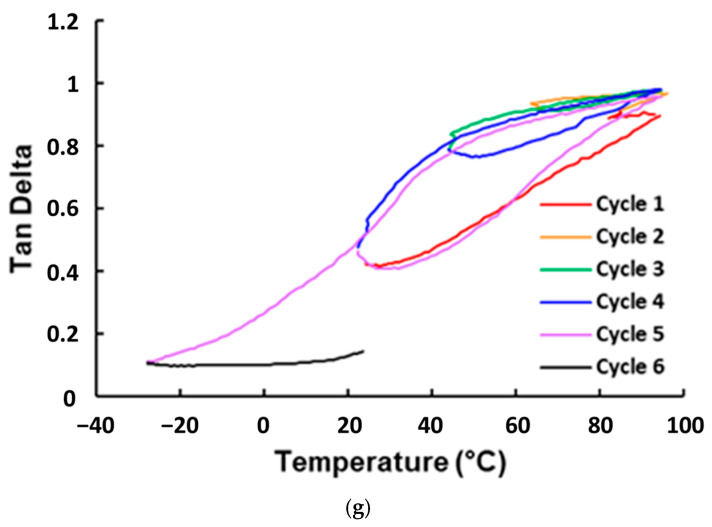
DMA results (Tan Delta-versus-temperature relationship in thermal cycling) of three-layered samples of vitrimer (**a**), TPU (**b**), EVA (**c**), STM (**d**), Blu Tack (**e**), five-layered sample of vitrimer (**f**), and three-layered sample of STM with one-directionally stretchable fabric (**g**) (sample length direction is along non-stretchable direction).

The Tan Delta-versus-temperature relationships in thermally cyclic DMA tests of all laminated samples are presented in Figure 7. It appears that except the laminated Blu Tack sample, the Tan Delta of all three-layered samples with two-directionally stretchable fabrics (Figure 7a–e) increases after the first heating process. On the other hand, while the Tan Delta of the laminated Blu Tack sample is always around 0.33 in the subsequent thermal cycling, Tan Delta may drop gradually or dramatically upon cooling in all other samples. Refer to the DSC results in Figure 4, where crystallization is mostly associated with the drop in Tan Delta upon cooling.

**Figure 8:**
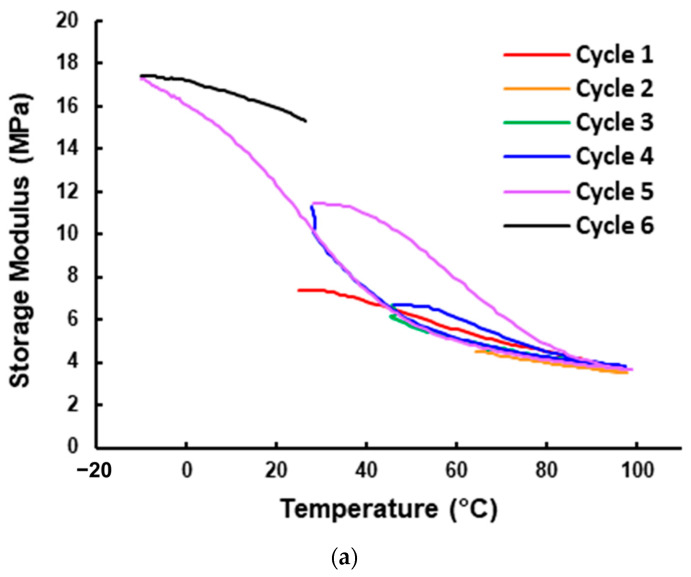

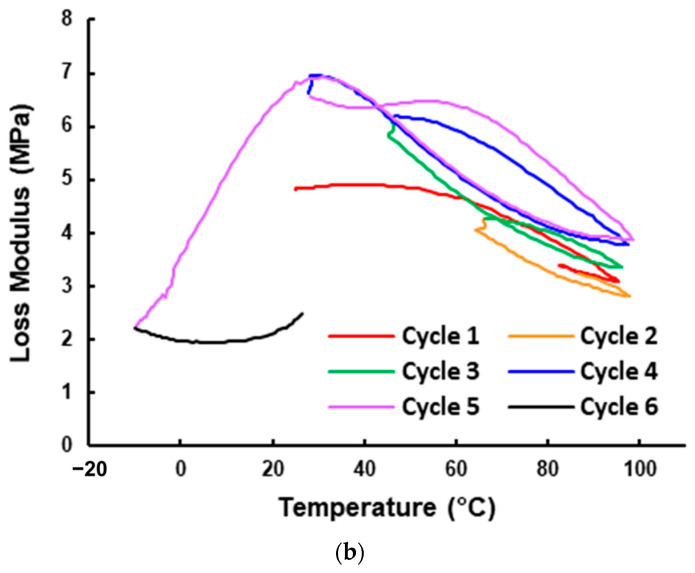
Storage modulus- (**a**) and loss modulus-versus-temperature relationships (**b**) of laminated STM sample in thermal cycling (same testing result as Figure 7d).

**Figure 9:**
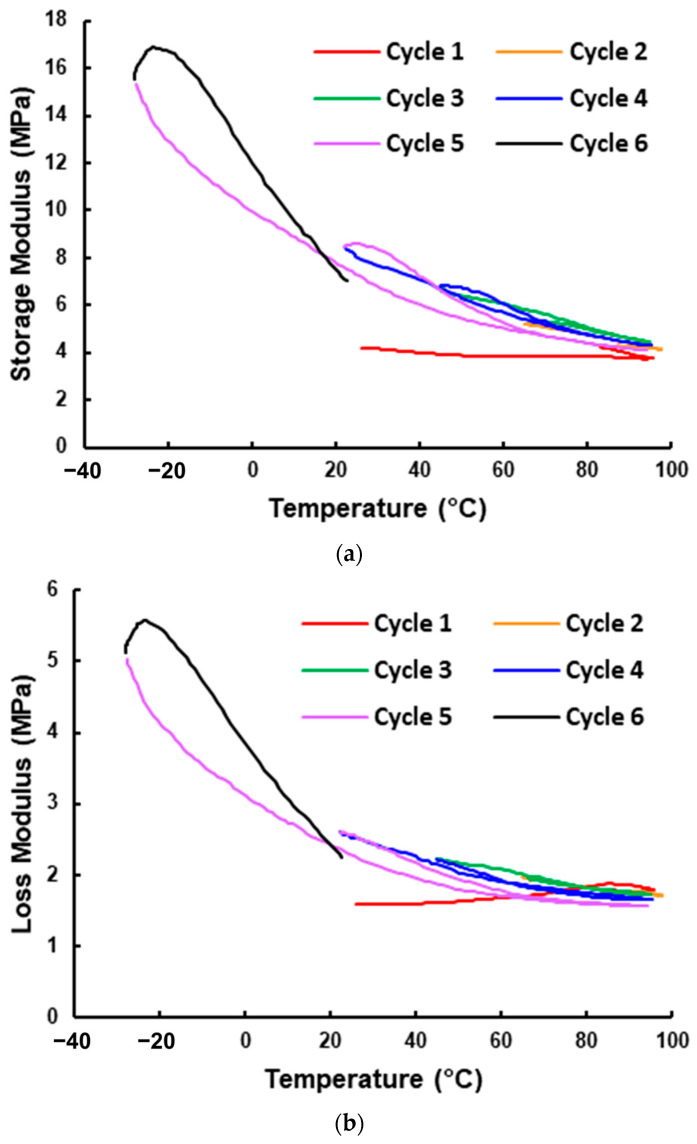
Storage modulus- (**a**) and loss modulus-versus-temperature relationships (**b**) of laminated Blu Tack sample in thermal cycling (same testing result as for Figure 7e).

As for the laminated STM sample (Figure 7d), in which there is not any apparent fluctuation in its DSC result upon heating and cooling, as shown in Figure 4d, the variation in the storage modulus and loss modulus upon thermal cycling can be easily spotted in Figure 8 (same experimental data as for Figure 7d). On the other hand, the fluctuation in the storage modulus and loss modulus of the laminated Blu Tack sample upon thermal cycling follows about the same pattern as shown in Figure 9. Consequently, the variation in Tan Delta upon thermal cycling after the first heating process (as shown in Figure 7e) is the least among all laminated samples, and there is no TDP.

While a TDP is apparent in all three-layered samples except the laminated Blu Tack sample, after the first heating, the laminated vitrimer sample appears to have the most stable plateau within around 20 °C to 90 °C with a Tan Delta value around 0.61. The Tan Delta value of the laminated vitrimer sample is more stable than that of pure vitrimer (refer to Figure 6a), since after melting upon further heating the reversible cross-linking gradually disappears. Hence, it can be concluded that a softer elastic part results in a higher Tan Delta value. According to Figure 4a, upon cooling to 20 °C, vitrimer starts to crystallize. Thus, the corresponding Tan Delta value starts to drop.

Upon thermal cycling, the Tan Delta value of the laminated TPU sample increases from 0.6 to 0.8 (Figure 7b). Although according to Figure 4b, crystallization of TPU starts at about 30 °C, the Tan Delta value starts to drop only upon cooling to below 0 °C. The Tan Delta value of the laminated EVA sample is mostly below 0.4 and the TDP is limited to between 40 °C and 95 °C, although according to Figure 4c, its crystallization peak temperature is around 60 °C. Such a kind of difference between the crystallization temperature and the temperature that the Tan Delta value starts to drop, observed in both the laminated TPU and EVA samples, is most likely due to time delay in crystallization.

Although the DSC result in Figure 4d does not show any apparent transition, Figure 8b, which reveals the variation in the loss modulus upon thermal cycling, clearly indicates the difference below and above around 20 °C. Of course, it is not within the scope of the current study to explore what the underlying mechanism is. As Figure 7d reveals, the laminated STM sample has the highest TDP after a couple of thermal cycles, reaching around 1 between about 40 °C to 95 °C. Even at the experiment starting point of 20 °C, its Tan Delta is already above 0.6. As for the other material with no apparent transition in its DSC result, Blu Tack, its laminated sample has a relatively stable, but lower Tan Delta value, between 0.3 to 0.4 within the whole tested temperature range (Figure 7e).

In comparison with the three-layered laminated vitrimer sample, five-layered laminated vitrimer sample shares the same trend in its Tan Delta-versus-temperature relationship upon thermal cycling. However, the corresponding Tan Delta value of the TDP is only between 0.4 and 0.5 (Figure 7f).

If the length direction of the three-layered STM sample is the non-stretchable direction of the one-directionally stretchable fabric, Figure 7g shows that the TDP is less flat, but seems more stable (with apparent hysteresis), when compared with the result of the three-layered STM sample with two-directionally stretchable fabric (Figure 7d). Its Tan Delta increases with the increase in temperature, reaching about 1 at 95 °C.

## 4. Discussion

According to Equation (1), a higher Tan Delta requires a higher E″ and lower E′, and a TDP is meant for about a constant E″/E′ value. As a special case, a constant E″ and a constant E′ over a long temperature range result in a TDP within a long temperature range.

Upon heating to above the melting temperature of a thermoplastic, the E′ of the polymer decreases, while its E″ increases, and thus the material starts to become easier to flow and the corresponding Tan Delta increases dramatically upon further heating.

Additional experiments were carried out to investigate the fluctuation in Tan Delta upon heating in two other types of configurations, namely the matrix-inclusion type of composite and cross-linked vitrimer.

### 4.1. Matrix-Inclusion Type of Composites

In the case of the matrix-inclusion type of composites, polydimethylsiloxane (PDMS, elastic silicone with a shore hardness about 45A [21]) was used as the matrix, while two types of polyurethanes (PUs), one being a thermoplastic PU (namely, TPU 265; refer to [18] for its details) and the other being a vitrimer-like PU (TPU 262; refer to [15] for its details), were used as the inclusions. The samples were prepared in a similar way as reported in [21]. The diameter of these inclusions is about 10 ~ 20 μm (similar to that reported in [21]).

**Figure 10:**
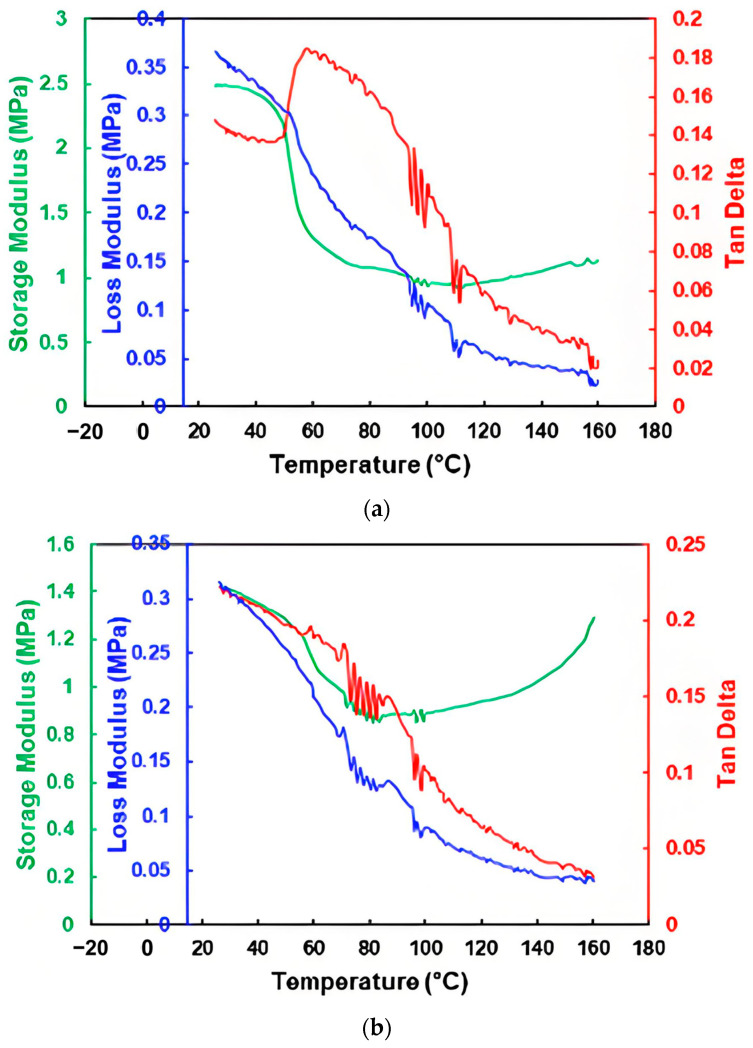
DMA results of composites produced with 60 vol% of PDMS and 40 vol% of vitrimer-like PU (**a**) and 60 vol% of PDMS and 40 vol% of TPU (**b**).

DMA results of both composite samples are presented in Figure 10. Upon heating, there is an apparent peak at around 60 °C in Tan Delta before it drops continuously in the PDMS–vitrimer PU sample (Figure 10a), while for the PDMS–thermoplastic PU sample, its Tan Delta drops continuously (Figure 10b). The maximum Tan Delta of both samples is always less than 0.4. A very weak bonding between silicone and inclusions in this type of composites reported in [21] is the reason for their relatively higher loss modulus at room temperature.

The volumetric fraction of the inclusions in a matrix-inclusion type of composite is limited to be less than 50 vol%. According to [22], 40 vol% of inclusion is about the upper limit in order to avoid the contact of spherical inclusions. Hence, a higher volumetric fraction of matrix (hence a much higher storage modulus) prevents Tan Delta being higher.

### 4.2. Cross-Linked Vitrimer

The cross-linking of vitrimer is an alternative to the above matrix-inclusion type of composite. After cross-linking, there are two networks in the material; one is permanent, while the other is reversible. The cross-linked vitrimer may also be considered as a kind of composite, in which there are two networks. At higher temperatures, the permanent network serves as a 3D mesh, so that even after severe quasi-plastic deformation, the sample is still able to return its original shape due to the shape memory effect.

**Figure 11:**
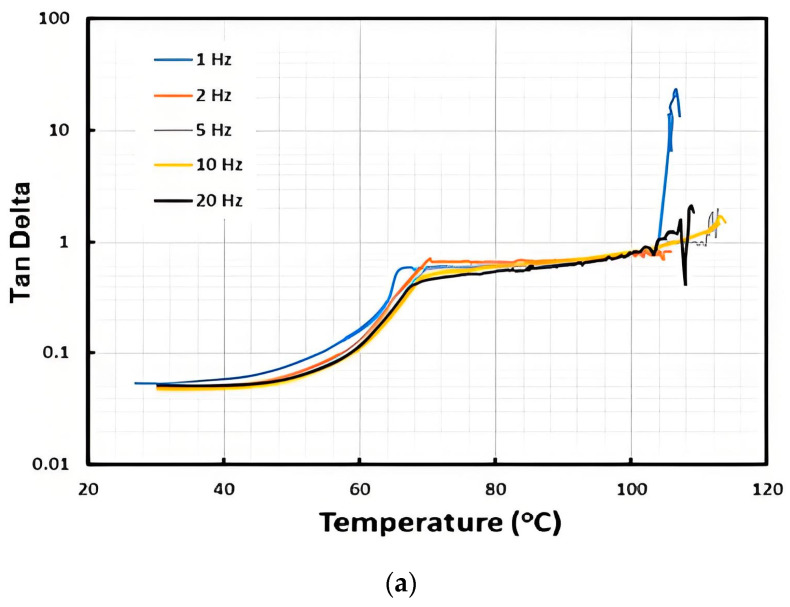

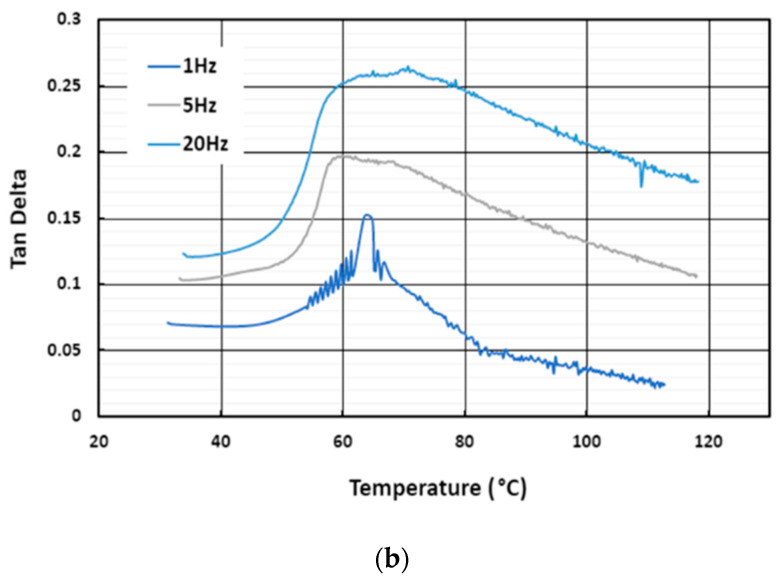
Tan Delta of the original (**a**) and cross-linked (**b**) vitrimer-like PU at different frequencies.

Dicumyl peroxide (DCP) is a commonly used initiator for cross-linking of polymers. TPU 262A, which is vitrimer-like and very much similar to TPUA-123-H1111A used in this study [15], was cross-linked with 2 vol% of DCP. The results of DMA testing, conducted at various frequencies, for samples with and without cross-linking, are illustrated in Figure 11. In Figure 11a, which is for the samples without cross-linking, an apparent TDP at all tested frequencies could be seen. On the other hand, In Figure 11b, which is with cross-linking, an apparent peak appears at around 65 °C in all tested frequencies. The actual shape of the peak and magnitude of Tan Delta depend on the frequency. In the case of 1 Hz, which results in the highest Tan Delta in all three tests, the maximum Tan Delta is less than 0.275.

**Figure 12:**
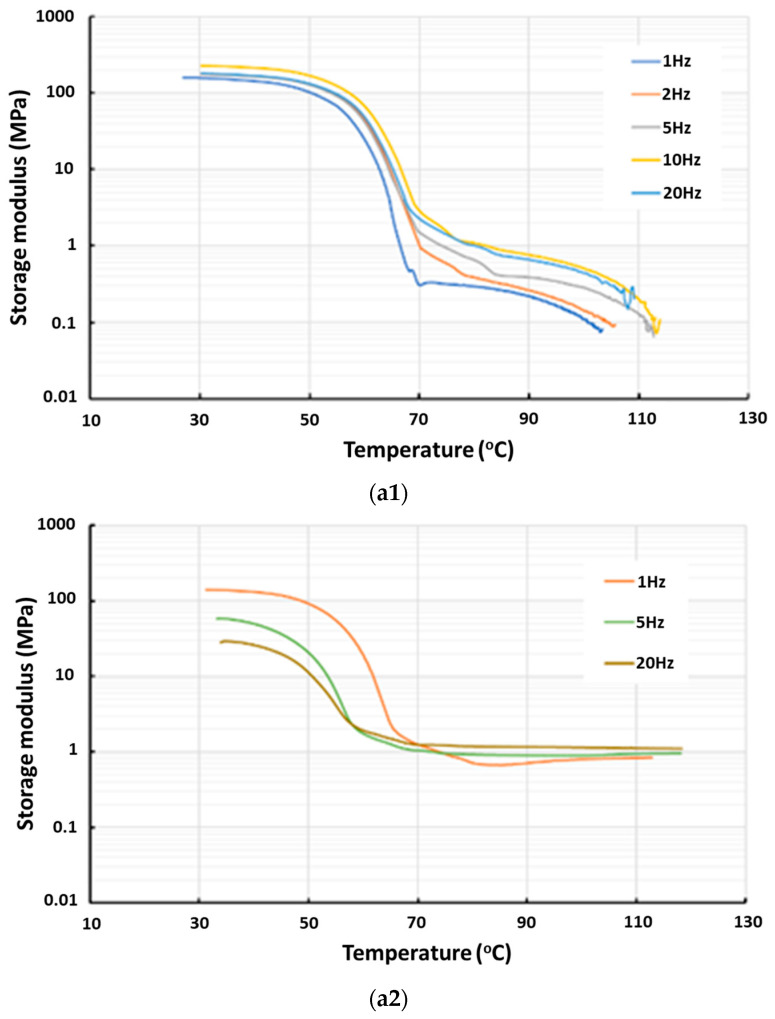

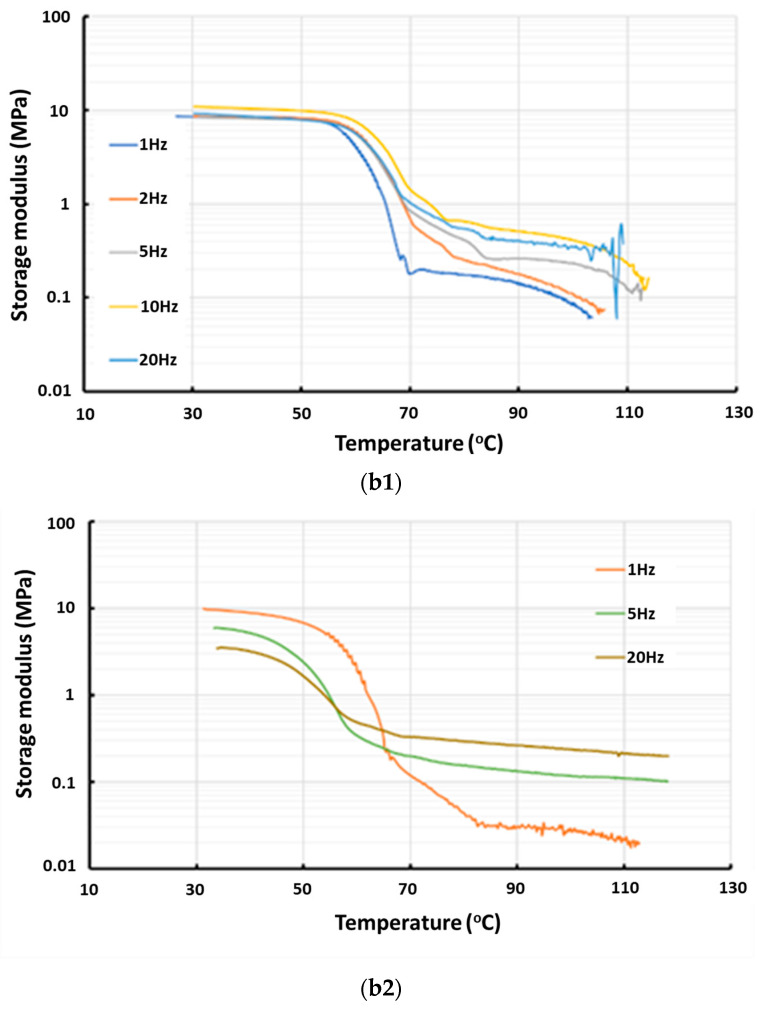
Storage modulus (**a**) and loss modulus (**b**) of vitrimer-like PU before (1) and after (2) cross-linking. (Data from same tests as in Figure 11).

In Figure 12, the storage modulus and loss modulus upon heating of the vitrimer with and without cross-linking are plotted. While the storage modulus of the original vitrimer drops continuously after a remarkable decrease at around 60 °C, after cross-linking, its storage modulus is about constant from around 80 °C. The continuous drop upon further heating in the original vitrimer is a result of the gradual disappearance of the reversible cross-linking [14,15]. On the other hand, in the cross-linked vitrimer, the permanent network keeps its storage modulus as a constant after melting. Apparently, this permanent cross-linking is a lot stronger than the reversible cross-linking.

The loss modulus of both cross-linked and non-cross-linked samples exhibits many similarities to the storage modulus, with the exception that the loss modulus of the cross-linked vitrimer gradually decreases with additional heating.

It appears that after cross-linking, the resulting Tan Delta is similar to that of ordinary semi-crystalline polymers, i.e., with an apparent Tan Delta peak at around its melting transition range.

### 4.3. Future Remarks

**Figure 13:**
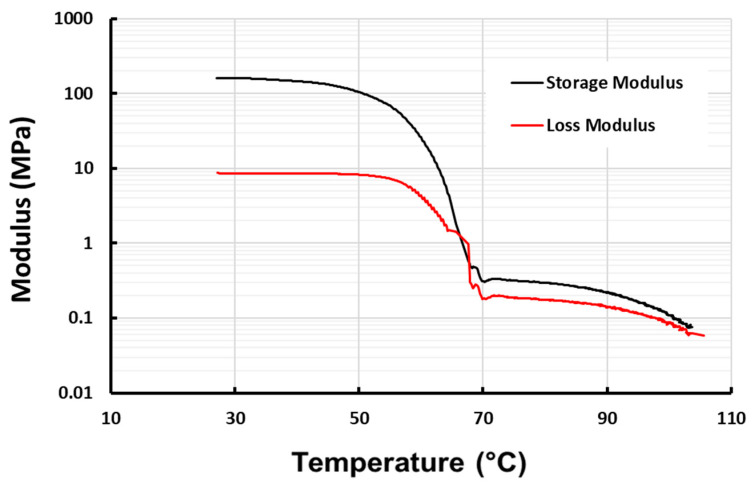
Storage modulus and loss modulus of three-layered vitrimer sample in the first heating process. (Same test as for Figure 7a).

**Figure 14:**
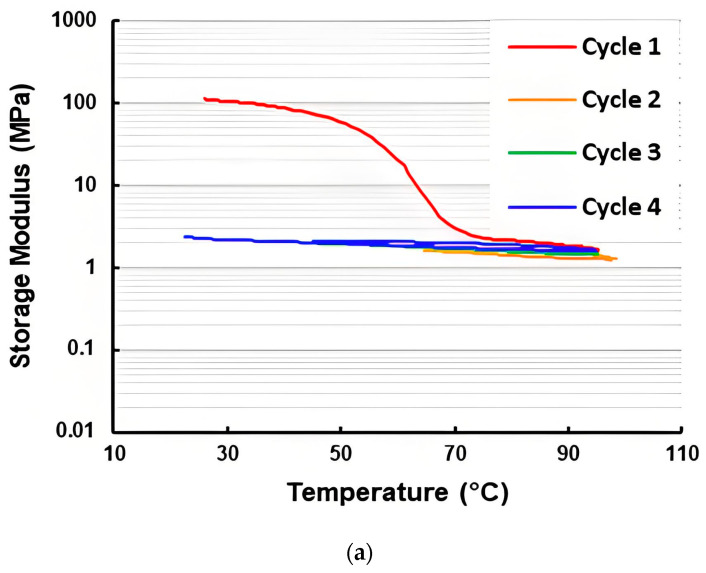

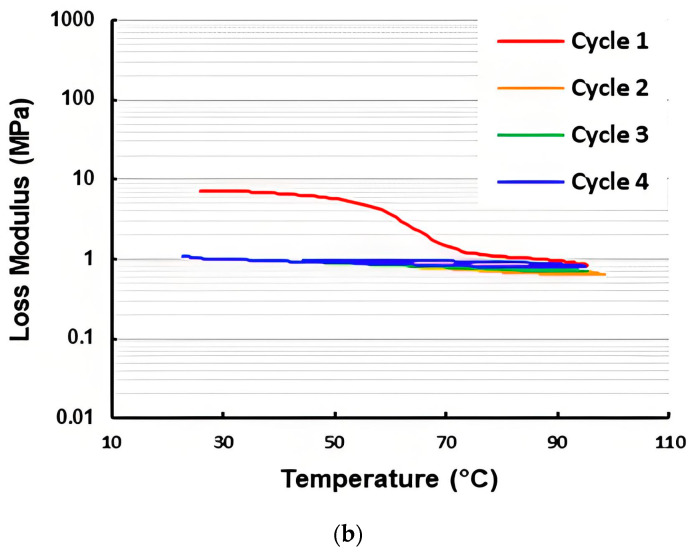
Storage modulus (**a**) and loss modulus (**b**) of five-layered vitrimer sample in the first four cycles. (Same test as for Figure 7f).

Based on the same experimental data for Figure 7a, the corresponding storage modulus and loss modulus of the three-layered vitrimer sample in the first heating process are plotted in Figure 13. In Figure 14, the storage modulus and loss modulus of the five-layered vitrimer sample (refer to Figure 7f for the corresponding Tan Delta) in the first four cycles are presented. Although the curves of the storage modulus and loss modulus for both three-layered and five-layered vitrimer samples look similar, upon heating to over 70 °C, both the storage modulus and loss modulus of the three-layered vitrimer sample are lower than that of the five-layered vitrimer sample. A closer look reveals that above 70 °C, the storage modulus curve and loss modulus curve of the three-layered vitrimer sample become more and more close to each other upon heating, while the storage modulus curve and loss modulus curve of the five-layered vitrimer sample are always not close. Thus, the TDP of the three-layered vitrimer sample has a higher Tan Delta value (but lower than that of the pure vitrimer sample, i.e., without elastic fabric, as shown in Figure 6a), despite the volumetric fraction of vitrimer being lower in the three-layered vitrimer sample.

This finding cannot be straightforwardly explained by Equation (1). It may be associated with the interaction between the elastic layer and polymer, and it reveals the influence of the actual layered architecture on the resulting Tan Delta in the TDP, hence providing an approach to tailor the Tan Delta of the TDP.

**Figure 15:**
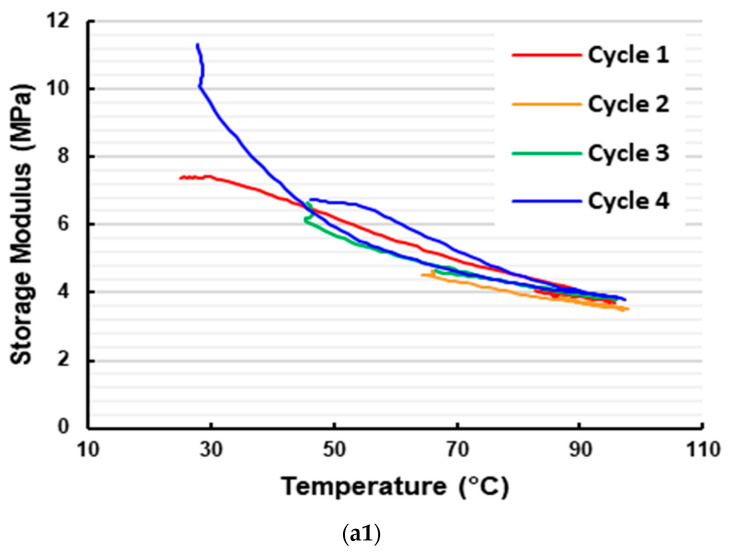

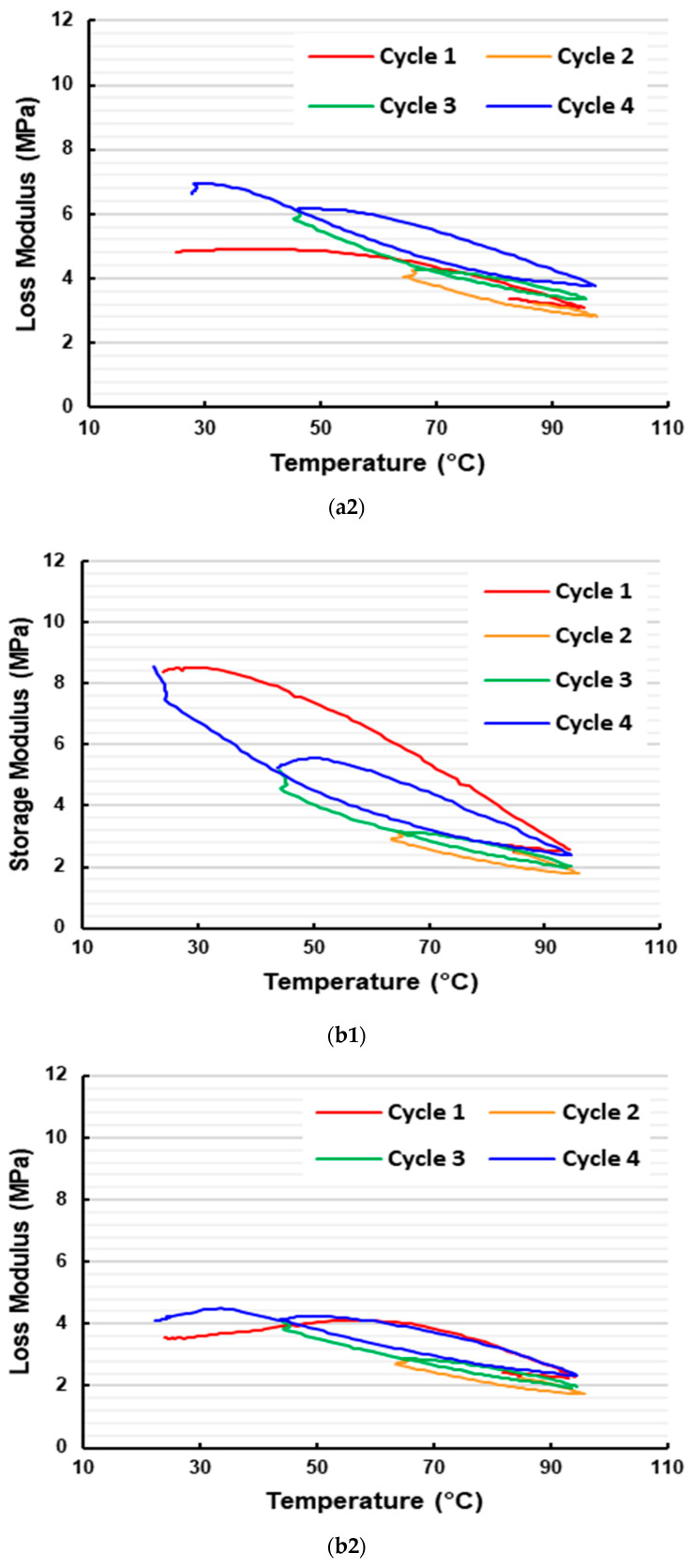
Storage modulus (**a**) and loss modulus (**b**) of three-layered STM samples in the first four cycles. (1): Stretchable direction (same test as for Figure 7d); (2): non-stretchable direction (same test as for Figure 7g).

In Figure 15, the storage modulus and loss modulus of two types of three-layered STM samples reported in Figure 7d,g in the first four cycles are presented to compare the effects of the stretchability of the fabrics. Although no apparent transition from the DSC result of this STM (refer to Figure 4d) could be observed, its storage modulus and loss modulus do drop slightly and gradually upon heating according to Figure 15.

STM is featured by its shear-hardening function, which means that it is easy to flow under quasi-static loading and becomes tougher under impact. Hence, under a loading frequency of 1 Hz, the sample coated with a relatively harder fabric (i.e., along the non-stretchable direction) appears to be more stable. With a softer fabric (i.e., along the stretchable direction, which is more viscous-elastic), laminated TPU, EVA, Blu Tack, and STM samples are similarly unstable upon thermal cycling, while the stability of laminated vitrimer samples is a lot better due to the reversible cross-linking.

Higher hysteresis along the non-stretchable direction of the 1D stretchable fabric as revealed in Figure 5 should be the main reason for the higher hysteresis in the storage modulus of the laminated STM sample with non-stretchable fabric upon thermal cycling in Figure 15(a2). On the other hand, high viscous-elasticity along the stretchable direction of the fabric may contribute to a slightly higher Tan Delta value.

## 5. Conclusions

A few laminated materials with a polymer layer in-between two elastic fabric layers were fabricated and their corresponding Tan Delta values upon thermal cycling were investigated via the DMA test. The materials used for the polymer layer include vitrimer, TPU, EVA, STM, and Blu Tack. The DSC test was carried out to reveal if there is any transition in these materials upon thermal cycling. The elastic fabrics are 1D and 2D stretchable spandex. Some additional samples were also produced to investigate the influence of a different multiple-layered architecture, the matrix-inclusion type of composite, and the cross-linking of vitrimer.

TDP was observed in all these layered samples, which confirms that the concept of layered architecture is a convenient approach to achieve a TDP. On the other hand, both the matrix-inclusion type of composite sample and cross-linked vitrimer sample do not demonstrate the TDP phenomenon.

Due to the reversible cross-linking, laminated vitrimer samples have the most stable Tan Delta value in the TDP. Furthermore, the actual Tan Delta value within the TDP is adjustable via tailoring the multiple-layered architecture. In the particular configuration of three-layered architecture, Tan Delta is around 0.6 from 20 °C to 100 °C, while in the configuration of five-layered architecture, it is around 0.45. This appears to be at odds with the definition of Tan Delta (=E″/E′).

On the other hand, the highest Tan Delta value for the same three-layered architecture (around 1.0) is observed in the STM samples. In the case that the so-called non-stretchable direction of the fabric is the sample length direction, the fluctuation in the Tan Delta value upon thermal cycling is remarkably reduced, while strong hysteresis is observed, which is due to significant hysteresis of the fabric along this non-stretchable direction.

Although this study offers a convenient method for achieving a TDP in engineering applications, there are challenges in comprehensively understanding the underlying mechanism for the TDP based on conventional theory. Specifically, a higher Tan Delta value is observed in a three-layered sample rather than a five-layered one, which may be associated with the interaction between the polymer and elastic layer. The customization of the Tan Delta value for the TDP is another aspect that needs exploration before integration into engineering applications.

## Figures and Tables

**Table 1:** Procedure of cyclic DMA test.

Cycle Number	Heating Process		Cooling Process		Ramping Rate (°C/min)
	Start	Finish	Start	Finish	
1	25 °C	95 °C	95 °C	80 °C	10
2	80 °C	95 °C	95 °C	60 °C	10
3	60 °C	95 °C	95 °C	40 °C	10
4	40 °C	95 °C	95 °C	20 °C	10
5	20 °C	95 °C	95 °C	−30 °C	10
6	−30 °C	25 °C			10

## Data Availability

Data are contained within the article.
